# Women’s experience of pain during childbirth

**Published:** 2010

**Authors:** Nastaran Mohammad Ali Beigi, Khadijeh Broumandfar, Parvin Bahadoran, Heidar Ali Abedi

**Affiliations:** * Department of Midwifery, School of Nursing and Midwifery, Isfahan University of Medical Sciences, Isfahan, Iran; ** Associate Professor, Khorasgan Branch, Islamic Azad University, Isfahan, Iran

**Keywords:** Women’s experience, pain, delivery pain

## Abstract

**BACKGROUND:**

Labor pain is one of the most severe pains which has ever evaluated and its fear is one of the reasons women wouldn’t go for natural delivery. Considering different factors which affect experiencing pain, this study aimed to explain women’s experiences of pain during childbirth.

**METHODS:**

This was a qualitative phenomenological study. The study population was composed of 14 women in 6 weeks post-partum period of natural delivery. The data were collected by interview. The data were analyzed by Colaizzi’s seven-stage method.

**RESULTS:**

After analyzing the interviews, four main categories were extracted: the nature of delivery pain, the related factors in labor pain, the results of labor pain, and the perception of caseworkers.

**CONCLUSIONS:**

Assessing the women’s experiences can be useful in giving better care. It helps understand the delivery pain phenomenon. Positive aspects of delivery pain must be strengthened and its negative aspects must be reduced as much as possible to create a suitable vision towards it.

Undoubtedly delivery is a painful experience for all of the women except a few of them. The labor pain results from some physiological-psychological causes. If the woman looks at the pain with a psychological view her feeling toward it would be changed.^1^

Having an abnormal pregnancy, low knowledge and bitter experience of the previous pregnancies can increase the labor pain while a normal pregnancy, having self esteem, pleasure and relaxation can decrease it and hence make your delivery favorable and if the woman would be pleased and relaxed, without facing real pains, her pain tolerance threshold would be increased.^2^

Pain is a phenomenon that hasn’t been understood and discovered completely yet and its clinical measurement could be really difficult. The individual experiences’ of the pain would be considered as a reliable source for its comprehension and only the individual can explain her experience. The pain is what was experienced by someone and it exits whenever she/he talks about it.^3^

In a study which conducted on 288 Swedish women, 28% of them evaluated labor pain as a positive condition and 41% of them considered it as the worst experience that they have.^1^

Fear of labor pain is one of the most important reasons that make women go for cesarean section. In a study on reasons for tendency to the cesarean section in Iran, fear of the labor pain was reported as 37.2%.^4^

The pain tolerance is the individual’s endurance and the acceptance of the pain in a specified range. It may differ in different people and could be influenced by the individual’s physical, psychological and cultural conditions.^3^ Reaction to the pain is also different in each person. Culture, gender, religious beliefs, and age can affect one’s assumption of pain and their reaction to it. For instance in Korean culture it is really important for the women to be quiet during delivery so they will not make their family ashamed. But European Americans show a wide range of reactions toward pain.^5^ Historical documents show that some societies have accepted pain as a part of their life and considered it as a fundamental element for growth and spiritual promotion.^6^

Anyway, the pain accompanies with the tension reactions and for resolving it, therapeutic and aggressive interventions are needed.^7^ Considering that the factors affecting pain experience include the age, prediction of the pain, gender and culture^8^, so the proper therapeutic and psychosocial interventions should be done for each person based on her/his culture, psychosocial strengths and weaknesses and individual needs.^9^

Also, considering that pregnancy and delivery are physiologic situations that can cause different feelings in a person^10^ and demographic data can influence women’s access to information and programming for managing their delivery.^11^ Also, by considering many cultural and social differences between Eastern and Western societies and certain beliefs that women in our country have about birth and delivery, the researcher decided to study women’s experiences of delivery pain.

## Methods

The Subject of this study was the women’s experiences of the labor pain and it considered as a phenomenological study for its issue.

For sampling, the researcher studied all the mothers’ medical files and then got the approval of those who had the criterion to be included in the study, which was the ability to explain their experience. Then, after setting an appointment, they met in the hospital or at home 6 weeks after delivery. The study population was consisted of mothers who had natural delivery and was in the 6^th^ week after delivery. Therefore research environment was the midwifery wards of Isfahan city hospitals and participants’ houses in 2008. The interviews were conducted in a quiet place without the interruption of the staffs or family members. All of the interviews were recorded completely and then prepared as the manuscript, if any confirmation was needed the researcher called or visited participants again, then words or phrases which were related to the phenomenon were distinguished and desired codes were extracted from the manuscript. After 14 interviews data saturation was obtained.

The validity and reliability of the study was based on the four factors (real value, practicality, stability, and being based on reality) suggested by Guba and Lincoln.^10^

Real value was obtained by returning to the participants and getting their confirmations for the resulted manuscript. Researchers tried to obtain practicality by describing the study environment and observed processes and also by selecting participants from a wide range of age and social background. Stability was obtained when the participants gave similar and consistent answers to a question which was asked differently. In this study, it was tried to avoid any prejudice about the studied phenomenon before and after the interviews, so this study became based on the reality.

For data analysis, Colaizzi’s method was used. First, for sympathizing with participants all of the manuscripts were read and important phrases were extracted. In the next step the meaning of each important phrase were described. Then codes were categorized and these categories were referred to initial protocols for confirming their validity. In the next step results were combined to this study as perfect description for the phenomenon and then were reviewed to obtain clear meaning and avoid any ambiguity. At the end the results were referred to participants for the confirmation.

## Results

This study was done by the interview with 14 help seekers who were hospitalized in the postpartum wards of Isfahan hospitals. Among all of the participants, 9 of them had their first delivery, 4 and one had their second and fifth delivery, respectively and all of them were aged between 18-35 years.

After analyzing and extracting meaningful codes, written interviews were categorized into 4 main concepts:

1. Nature of labor pain
2. Related factors in labor pain
3. Results of labor pain
4. Perceptions of help-seekers

### 

#### Nature of labor pain

Nature of labor pain is consisted of two sub-concepts of “severity and type of the pain” and “feelings accompanied with labor pain”. Help seekers’ experience of severity and type of the pain was described as, “delivery pain is really hard to endure, it is not similar to other pains … the most severe pain that I’ve ever tolerated was labor pain”, told by one of the participants.

And also “its pain is unbearable and indescribable” was said by another participant.

To describe the type and location of the pain another participant said, “I had pain during my menstruation; its pain was like that but 10 times more”.

One of the unique aspects of labor pain is that this physiologic process is not simple. Based on participants’ experiences labor pain is accompanied with some special feelings; one of the participants said, “labor pain is the sweetest pain in the world, I love it so much, of course it is hard to endure but it is sweet”.

Another participant mentioned, “I am worry because I have delivered a girl. My husband said that this baby must be boy. My parents are also worry because I have delivered a girl because my husband always said that he wanted a boy…I am so worried that when I leave here my husband won’t pay any attention to me”.

Another feeling is fear accompanied with labor pain which another participant described it so, “I was afraid, I was so scared that I couldn’t talk; only God knows what will happen at the end”.

#### Related factors in labor pain

Related factors to labor pain are consisted of two concepts of “factors which increase pain” and “factors which decrease pain and make it tolerable”. One of the participants experience, “The pain would increase each moment… the pressure of the baby’s head make the pain worse”.

Other factors which increase the pain are described as below:

”I was alone in a room and I was so scared when the pain started, I like that all doctors and nurses come to me and hold my hand when they hear I scream so much, if they hold my hand I would relax. Loneliness increased my pain”, said one of the participants.

Another participant believed that, “I was really bothered at the time of examination, one was going and another one was coming. They said that the examination should be done in the middle of the pain. My pain was increased during the examination. Every hour someone came for the examination. I request them to finish it but they wouldn’t accept at all and they said each one must have examined for herself, examination increase the labor pain…”

Based on the participants’ descriptions, there are some factors to decrease the labor pain like “support of the treatment personnel”.

One of the participants said, “if the nurse is mild and warm hearted you will feel less pain” and another participant believed that “when the nurses talk about the time of my delivery, I felt more comfortable and I could tolerate the pain better”.

As it could be understood from the participated mothers’ statements, their subjective beliefs were one of the effective and important factors in decreasing the labor pain. Regarding this matter one of the participants said, “I knew that my baby is a boy, I had heard that delivering a girl is more painful but a boy is easier and its pain is sweet. I thought that because my baby is a boy I would feel less pain and if the baby was not a boy the pain was so much more”.

Many of the participants mentioned the importance of the religious thoughts and beliefs in decreasing the labor pain, “God always help. God is great, I really believe in God. The more you are in pain, the more you call God and God would help you more. God never leave his creatures alone, God helped me to endure the pain”, One of the participants.

#### Results of the labor pain

The concept of the results of the labor pain has concluded from early and late reactions to the labor pain. So many authors have written about special importance of social-cultural factors in pain related beliefs and how to react to it.

Another participant said, “When the pain increased, I begin to cry. I was crying because of the pain. But the participant number 5 had a different reaction toward pain, “The pain was increasing and nothing could decrease it, I could not do anything, so I tolerated it because I knew it would be over soon”.

And another participant said, “I would suggest everybody to go for cesarean section”.

#### Perceptions of caseworkers

Going through natural delivery with all of its difficulties and sweet moments cause different perceptions in different help seekers. The concept of help seekers’ perceptions is derived from their experiences in the field of quality of health care, training and consulting, treatment expenses, family support and natural delivery.

As participants described, “Behavior of the delivery room’s personnel is not good. I was alone in a room and nobody answered me when I cried out. You must tolerate all of the pain until you are going to labor, one of the participants.

There are some concerns about treatment expenses. One of the participants has described it so, “I didn’t want to use pain killers in my delivery because I had to pay 15 thousands To-man more. It was there just to get more money from people and they took it to everyone and told them that it costs you 15 to 20 thousands Toman. When you don’t have the money you must tolerate the pain, if I had a better financial situation I would go to a better hospital. When someone is wealthy, she is more comfortable always”.

And another one believed that, “Women’s delivery must be recorded and then showed to their husbands so that they would know how difficult it is and how babies are being delivered. I only wish he had heard me scream because men don’t understand this subject. They always disregard women’s rights. Then they say we want a boy, it’s hard to endure, it’s so bothering that my husband took care of me and took me to doctors hoping that the baby is a boy”.

Another participant said, “I would suggest everyone to go for cesarean section because during the surgery you are unconscious and would not understand anything and you would feel no pain but in natural delivery you can see with your own eyes that how much difficulty you are going through. But if the doctor is good, I would say go for natural delivery”.

And another participant made this comment about natural delivery so, “Natural delivery is good… it’s better…you only feel pain for 2 to 3 hours and after delivery there is no pain anymore but in cesarean only during the surgery you won’t feel no pain. After becoming conscious the pain starts, you can’t walk, you can’t move, you can’t even go to the bathroom”.

## Discussion

Results of this study showed that help-seekers gain special experiences during delivery. help-seekers’ descriptions of their experiences about the labor pain were categorized into four main concepts of the nature of the labor pain, the related factors to the labor pain, the results of the labor pain and the perceptions of help-seekers toward the labor pain.

Based on the presented experiences, labor pain has a special nature which includes pains characteristics regarding its type and location and also includes feelings which accompany pain. Emotional and mental features which were described by the participants are valuable experiences that should be taken into consideration because labor pain is one of the most severe pains that a woman should endure in her life. One of the unique aspects of the labor pain is that this physiologic process is not easy. Assessing the presented experiences reveal that labor pain is a complicated result of interactions between numerous mental and physiological factors so to control the pain, medical team should understand patient’s experiences about pain.

By analyzing help seekers’ experiences it was revealed that labor pain has a special aspect and that is being accompanied with different feelings and emotional states which is negative sometimes and mostly is positive and joyful; based on some of the participants’ statements this aspect made the labor pain a desirable memory. Also Kathleen (2006) in a study achieved that the labor pain can be enjoyable, relaxing and pleasure.^12^

Experiences showed that medical system and the family participations which were supporting the help seekers could decrease the pain and make it tolerable. Religious thoughts and some of the personal characteristics could also help the help seeker during this experience. But many of the actions in support system and personal characteristics could be bothering during labor pain and even make it worse and these factors were effective in making delivery a bad memory. Specific desired gender in husband’s attitude is one of these factors. In many Asian and African countries couples happiness after delivery is based on the gender of the baby but in European and North American countries where welfare status is different there is no certain priority for baby’s gender. In Asia and Africa, boys are in clear superiority.^11^

Another result of this study was the results of the pain in participants. Considering the characteristics and cultural essence of the individuals has a special importance in the way they face pain. One’s recognitions (beliefs, evaluations and expectations) about results of the labor pain and also her ability to face it shape her viewpoints and functions which can change her function and reaction toward pain. Unwillingness toward natural delivery and increase the number of cesarean sections in the country is a result of negative viewpoints that help-seekers have gained through their experiences and transmitted to the society. One of the reasons of fear of natural delivery is transmission of bad experiences and hearing horrible stories from women who experiences difficult natural deliveries before. This is one of the reasons that women with their first pregnancy prefer cesarean section.^13^

Therefore, considering that beliefs and view-points about each issue would be shaped based on transmission of experiences and social-cultural achievements. It is important to take the importance of these factors in making viewpoints and beliefs about labor pain in consideration.

Results of this study have somehow shown challenges in health care system, the family awareness and people’s beliefs about labor pain which could be used to improve the quality of natural delivery and change negative approach toward the method of delivery, if it would be supported by more researches.

The Authors declare no conflict of interest in this study. The ethical committee approved the study.
